# Jointly Learned 3D Non‐Cartesian Sampling With Wave Encoding and Reconstruction for Neurovascular Phase Contrast MRI

**DOI:** 10.1002/mrm.70215

**Published:** 2025-12-08

**Authors:** Chenwei Tang, Brock W. Jolicoeur, James Rice, Caroline A. Doctor, Zaynab S. Yardim, Leonardo A. Rivera‐Rivera, Laura B. Eisenmenger, Kevin M. Johnson

**Affiliations:** ^1^ Department of Medical Physics University of Wisconsin Madison Wisconsin USA; ^2^ Department of Radiology University of Wisconsin Madison Wisconsin USA; ^3^ Department of Biomedical Engineering University of Wisconsin Madison Wisconsin USA; ^4^ Department of Mechanical Engineering University of Wisconsin Madison Wisconsin USA; ^5^ Department of Physics University of Wisconsin Madison Wisconsin USA; ^6^ Department of Medicine University of Wisconsin Madison Wisconsin USA

**Keywords:** deep learning, non‐cartesian sampling optimization, phase contrast, wave encoding

## Abstract

**Purpose:**

To develop accelerated 3D phase contrast (PC) MRI using jointly learned wave encoding and reconstruction.

**Methods:**

Pseudo‐fully sampled neurovascular 4D flow data (*N* = 40) and a simulation framework were used to learn phase encoding locations, wave readout parameters, and model‐based reconstruction network (MoDL) for a rapid 3D PC scan (2.25 min). Parameters were also learned for an otherwise identical scan without wave encoding. Prospective scans with and without wave sampling, time‐matched 3D radial, and reference 3D radial (5.65 min) were conducted in a flow phantom and 12 healthy participants. Flow rate, pixel‐wise velocity, and variability of maximum velocity ($\sigma_{v_{\max}}$) were compared.

**Results:**

In the phantom, learned wave scans provided accurate flow rates compared to flow probe values (0.170 ± 0.002 vs. 0.17, 0.152 ± 0.003 vs. 0.15, 1.838 ± 0.044 vs. 1.83 L/min) and showed high correlation with reference scan (slope = 0.97, *R* ^2^ = 0.99). In vivo, learned wave scans demonstrated reduced aliasing and blurring, and better small vessel conspicuity compared to scans without wave sampling and time‐matched 3D radial scans. The internal carotid artery (ICA) flow rate coefficient of variation (CV) and intraclass correlation coefficient (ICC) for learned wave scans were similar to reference 3D radial scans (CV = 6.569, ICC = 0.927; reference CV = 6.553, ICC = 0.910). Learned wave sampling demonstrated similar or lower $\sigma_{v_{\max}}$ in middle cerebral artery (MCA), basilar artery (BA), superior sagittal sinus (SSS), and most ICA segments than the longer reference scan.

**Conclusion:**

This work demonstrates feasibility, improved image quality and accurate flow measurements of learned wave sampling and MoDL reconstruction for 3D PC MRI.

## 1 Introduction

Cerebrovascular magnetic resonance imaging (MRI) is frequently used clinically for identifying abnormalities such as aneurysms, arteriovenous malformations (AVM), stenosis, and occlusions [1, 2], without requiring exogenous contrast agents or ionizing radiation. More recently, functional vascular imaging techniques have been developed to simultaneously provide angiographic and hemodynamic information. This includes methods based on the inflow of blood (3D quantitative time of flight [qTOF] [3], arterial spin labeling [ASL] [4]), and methods to directly measure blood velocities (e.g., phase contrast MRI (PC‐MRI) [5]). These methods provide the opportunity to analyze vascular changes beyond structural remodeling. In particular, PC‐MRI enables comprehensive evaluation of the vascular system, with the ability to resolve cardiac dynamics. The velocity information PC‐MRI provides can be used to assess blood flow rates, wall shear stress, and infer vessel compliance from pulse wave velocity and other hemodynamic metrics. Such information is not only useful for evaluating vascular abnormalities clinically but can also be used to understand vascular changes in normal aging [6, 7, 8, 9, 10] and the development of various pathologies that have connections to the vascular system, such as Alzheimer's [11, 12].

While cardiac‐gated 2D‐PC MRI is routinely collected clinically, cardiac‐gated 3D PC‐MRI (i.e., 4D‐Flow) is limited by long acquisition times due to multiple flow encoding directions and time required for cardiac resolved imaging. This is exacerbated by the need for high spatial resolution to depict small intracranial vessels. To obtain clinically feasible scan times, 4D‐Flow requires a high level of acceleration which is currently achieved with varying combinations of parallel imaging, compressed sensing (CS) [13], *k*–*t* acceleration schemes [14], and non‐Cartesian sampling [15, 16], all of which have benefits, limitations, and drawbacks. For example, with 3D radial, whole brain 4D‐Flow can be achieved with 0.7 mm isotropic/50 ms spatial/temporal resolution in under 6 min [15, 16]. It is robust to motion and amenable to CS as its undersampling artifacts are incoherent. However, it has a lower sampling and SNR efficiency and is more difficult to tailor to anisotropic fields of view (FOV) than Cartesian imaging. Wave‐CAIPI combines a non‐Cartesian readout with 2D CAIPI phase encoding pattern that is on a Cartesian grid [17, 18], enabling flexible FOV tailoring [18] and efficient *k*‐space traversing. However, Wave‐CAIPI requires tuning wave encoding parameters and has only been applied in body 4D‐Flow imaging [19]. The tuning of wave encoding parameters relies on simulations of voxel spreading, g‐factor maps, and relaxation. It can be challenging to connect these metrics to in vivo performance [18, 20]. It becomes increasingly complex when considering phase encoding sampling patterns aside from CAIPI (e.g., Poisson disc). In addition, the reconstruction time of these advanced acquisition methods is often on the order of hours, rendering them impractical for clinical applications such as fast screening. In these scenarios structural and time‐averaged flow rather than cardiac‐resolved hemodynamics may be sufficient for clinical decision making.

Deep learning (DL) presents as a powerful and pervasive tool that provides opportunities to improve both sampling and reconstruction aspects of MRI. Model‐based deep learning (MoDL [21]) and variational network [22] have been widely adopted for reconstruction, where data consistency is integrated with trainable networks. Recent studies have applied MoDL [23, 24] and other DL methods, such as deep image priors [25] and post‐reconstruction denoising [26, 27, 28] in PC‐MRI to improve image quality and velocity quantification in accelerated acquisitions. DL also offers methods for learning accelerated sampling strategies from prior data; however, these have not been applied to PC‐MRI. Direct optimization on the sampling pattern by maximizing the sampling efficacy [29], by mimicking the probability density function extracted from acquired fully sampled k‐space data [30], and by optimizing image quality [31, 32, 33] have been proposed. For example, LOUPE [33] and J‐MoDL [34] jointly learn Cartesian sampling and DL reconstruction, with U‐Net and MoDL, respectively. Sampling optimization can also be extended to non‐Cartesian, as in AutoSamp [35], which optimizes a 2D non‐Cartesian sampling pattern and MoDL reconstruction.

The extension of learned sampling and reconstruction to wave encoded PC‐MRI has potential but presents multiple challenges. First, gradients with respect to sampling coordinates were often tracked by the auto‐differentiation engine of PyTorch [36] or Tensorflow [37] through the non‐uniform fast Fourier transform (NUFFT) [35, 38] based on chain rule. Due to the approximation nature of NUFFT, this approach can be suboptimal and unstable. BJORK [39] and SNOPY [40] proposed analytical Jacobians of the NUFFT operator–matrix product and present a method to avoid this pitfall. Yet, these methods have not been widely applied to fully 3D sampling where there are challenges in collecting ground truth data and computational challenges associated with higher memory demands.

This work aims to investigate the use of DL to optimize 3D non‐Cartesian sampling trajectories and reconstruction. In particular, we develop a fast 3D time‐averaged intracranial flow imaging technique that can be used for clinical screening with simultaneously learned wave parameters, sampling coordinates and 3D MoDL reconstruction. As a comparison, sampling coordinates and MoDL reconstruction were also trained without wave encoding. We prospectively tested the learned wave sampling and MoDL reconstruction on a flow phantom and in human participants and compared to learned sampling without wave encoding and 3D radial sampling.

## 2 Methods

This work is organized based on training and evaluation stages: (1) training the sampling trajectories and reconstruction network with supervision and (2) prospective scanning with the learned sampling followed by reconstruction with the trained network. During training, multi‐channel non‐Cartesian k‐space data was simulated based on sampling from pseudo‐fully sampled volumetric images. The simulated k‐space data was subsequently reconstructed with MoDL and the image domain normalized root mean square error (NRMSE) to the pseudo‐fully sampled images was calculated. The loss with respect to both the sampling and MoDL parameters were tracked in minimizing the loss. Once trained, the sampling coordinates and MoDL were fixed and used for prospective testing in phantoms and human participant scans. This study was approved by the University of Wisconsin‐Madison Institutional Review Board. Written informed consent was obtained from all participants.

### 2.1 Training Data

Variable density 3D radial PC‐MRI scans were included from a standardized imaging protocol for neuroimaging studies at our institute [15]. Parameters include: TE/TR = 2.5/7.7 ms, *V*_enc_ = 80 cm/s, FOV = 220 × 220 × 220 mm^3^, resolution = 0.86 mm isotropic, 11 000 spokes, echo fraction = 0.75, scan time 5.65 min. We will refer to it as the “reference 3D radial” scan for the rest of this manuscript. Training included 20 healthy participant datasets acquired after the administration of Ferumoxytol (5 mg/kg) (acquired on 3T MR750, GE Healthcare and 32‐channel head coil, Nova Medical; healthy volunteers age 20–61 years, mean = 32 years, previously presented data [41]) and 20 participant scans without exogenous contrast agents from Wisconsin Alzheimer's Disease Research Center (WADRC) clinical cohort (acquired on 3 Signa Premier with 48‐ch head coil, GE Healthcare; age 50–78 years, mean = 66.8 years). Ferumoxytol enhanced 3D PC‐MRI scans were acquired with 5‐pt balanced flow encoding [42] while non‐contrast scans were collected with 4‐point flow reference encoding. Time averaged images were generated using an L1‐Wavelet regularized CS reconstruction using data from the entire acquisition without gating and binning. The regularization parameters empirically chosen based on perceived image quality (wavelet transform = DB‐4, iterations = 30 using FISTA [43] with 3% of coefficients removed at each iteration). Coil sensitivity maps were derived from the central k‐space area for each case and utilized in the optimization paradigm. These images were considered pseudo‐fully sampled ground truth images. Datasets were split with a 9:1 ratio for training/validation (18 post‐contrast + 18 non‐contrast scans in training, 2 post‐contrast + 2 non‐contrast scans in validation).

### 2.2 Wave Trajectory

Wave encoding uses sinusoidal gradients in $k_{y}$ and $k_{z}$ directions and a constant gradient in $k_{x}$ direction, resulting a helix trajectory. The coordinate $\omega =\left(k_{x},k_{y},k_{z}\right)$ of the ith point on a helix with radius r, cycles C, and rotation θ along $k_{x}$ axis is calculated by:

(1) $$k_{x}\left[i\right]=-C2\pi \left[\mathrm{ef}-\frac{1}{2}-\frac{\mathrm{ef}}{N_{\mathrm{RO}}}\;i\right]$$

(2) $$k_{y}\left[i\right]=r\sin\left(k_{x}\left[i\right]+\theta \right)$$

(3) $$k_{z}\left[i\right]=r\cos\left(k_{x}\left[i\right]+\theta \right)$$

where ef is echo fraction, $N_{\mathrm{RO}}$ is the total number of readout points. In theory, all wave parameters could be designed and optimized for each phase encoding position; however, this substantially increases the complexity to the optimization and pulse sequence implementation. To avoid this, we initialize all readouts to have the same wave trajectory with learnable helix rotation θ around the $k_{x}$ axis and scaling of the helix radius $s_{r}$. Thus, the trajectory is defined:

(4) $$k_{y}\left[j,i\right]=s_{r}\left[j\right]r\sin\left(t\left[i\right]+\theta \left[j\right]\right)+k_{\mathrm{yc}}\left[j\right]$$

(5) $$k_{z}\left[j,i\right]=s_{r}\left[j\right]r\cos\left(t\left[i\right]+\theta \left[j\right]\right)+k_{\mathrm{zc}}\left[j\right]$$

where j is the phase encoding index and $\omega_{c}=\left(k_{\mathrm{yc}},k_{\mathrm{zc}}\right)$, is the center position for each helix. One highlight of our work is that we are not learning a binary sampling mask [33, 34], but the sampling coordinates that are floating point number and are not confined on grids. Parameters in bold are learnable. The theoretical trajectory in Equations (4) and (5) were parameterized using the optimal‐time gradient method [44] to comply with the maximum gradient amplitude and slew rate. To avoid redesigning the helix to fit hardware limitations and maintain the same readout length, the helix radius can only be scaled down (i.e., $s_{r}\leq 1$). As the helix radius has a significant impact on the *g*‐factor [18], we chose a relatively large max radius ($g_{\max}=9\;\mathrm{mT}/m$) to minimize the g‐factor penalty. The echo fraction was set to 0.75 to match that of the 3D radial scan and minimize the echo time (TE) penalty for flow compensation and encoding. The number of cycles was fixed to 8 to have similar readout duration as the 3D radial scan (2.7 and 2.6 ms, respectively) and to suppress T2* blurring [18]. The actual trajectory deviates from the parameterized one due to hardware imperfections (Figure S6) and was measured using the thin slice method [45], with 32 slices in each polarity for each gradient axis. A single measured trajectory was shared among helices by scaling and rotation to generate the full coordinates ($N_{\mathrm{RO}}\times N_{\mathrm{PE}}\times 3$).

### 2.3 Optimization Model

**FIGURE 1:**
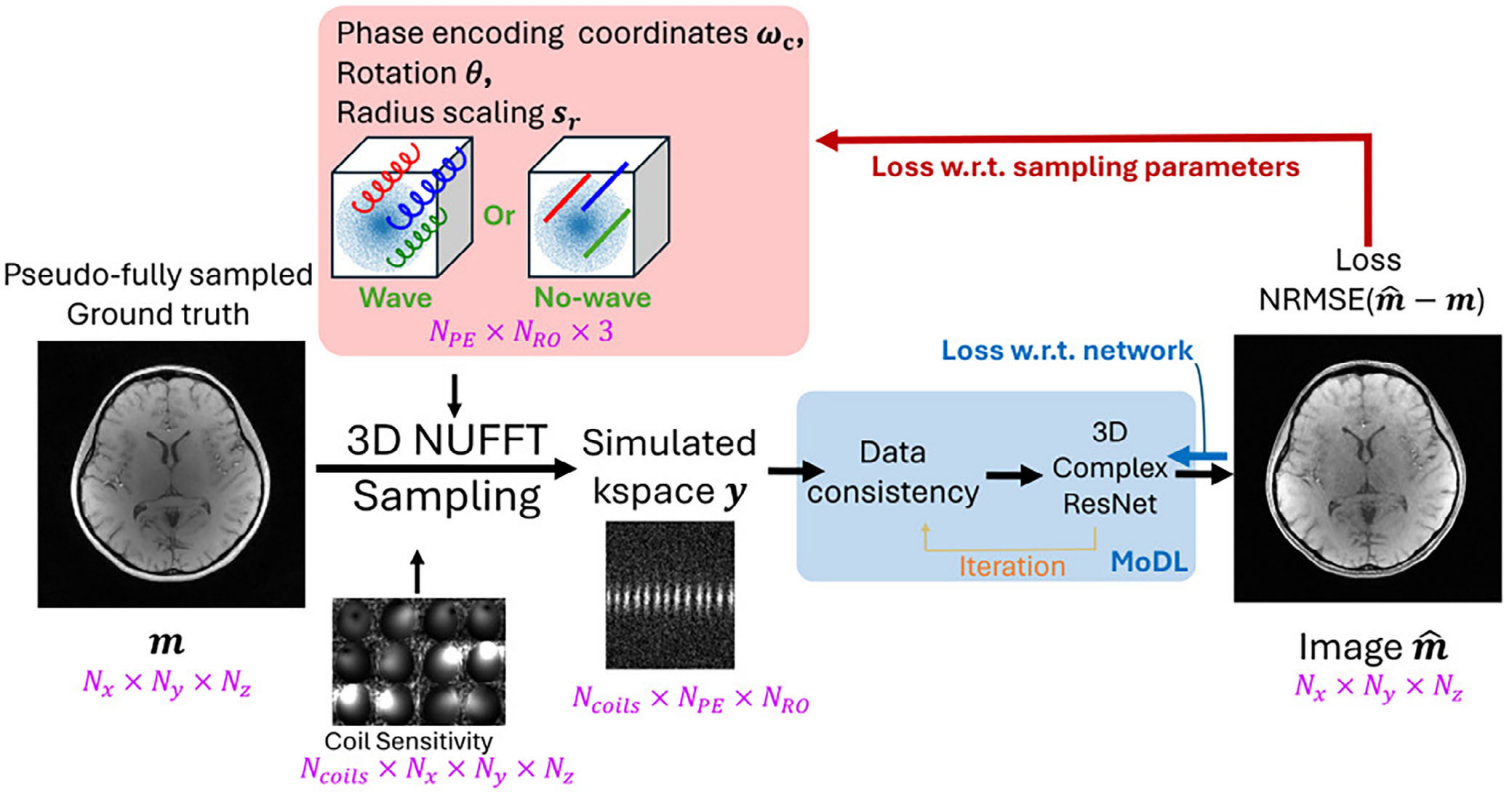
Schematic illustration of the optimization model. Multi‐channel 3D NUFFT was used to sample the pseudo‐fully sampled images at phase encoding coordinates $\omega_{c}$ coupled with wave or “no‐wave,” that is, straight‐line, frequency encoding. Simulated k‐space data is reconstructed with MoDL (blue box), which alternates between a proximal gradient data consistency term and a 3D complex ResNet regularizer. The phase encoding coordinates ω, wave rotation θ and radius scaling $s_{r}$, and the parameters in MoDL are optimized based on NRMSE loss. The red arrows highlight the path for loss backpropagation with respect to $\omega ,\theta ,s_{r}$ while the blue arrow tracks the loss with respect to the reconstruction model parameters. This framework can be memory‐demanding. The sizes of matrices are highlighted in magenta.

Figure 1 illustrates our end‐to‐end framework to learn both sampling trajectories and MoDL parameters. The general MRI acquisition can be expressed as:

(6) y=Am+ϵ=CFSm+ϵ

where the multi‐channel encoding operator A consists of coil sensitivity maps C, 3D NUFFT F and sampling scheme S and ϵ is random complex gaussian noise. y∈ℂ is the simulated multi‐channel k‐space data, m∈ℂ is the pseudo‐fully sampled ground truth image.

The work considers two cases for the sampling scheme (1) learnable phase encoding coordinates with straight‐line readout (referred to as “no‐wave” in the manuscript) and (2) learnable phase encoding coordinates with wave readout. Neither the coordinates of the helices center $\omega_{c}=\left(k_{\mathrm{yc}},k_{\mathrm{zc}}\right)$ nor the points along the readout were confined on Cartesian grids. Training is performed in a supervised manner, with the L1‐Wavelet reconstructed reference 3D radial scans as the target images. At each epoch, all training cases are gone through in a random order with no gradient accumulation. For each case, at the ith readout point on the jth helix $\left(k_{x}[i,j],k_{y}[i,j],k_{z}[i,j]\right)$, multi‐channel sampling is simulated on the pseudo‐fully sampled dataset using 3D NUFFT and the simulated k‐space data y is reconstructed with MoDL, which solves the MR reconstruction problem

(7) $$\hat{m}=\text{argmi}n_{m}{\left|\left|\mathrm{CFSm}-y\right|\right|}_{2}^{2}+\lambda R\left(m\right)$$

by alternating between a data consistency (DC) step

(8) $$z^{t+1}={\hat{m}}^{t}-2\alpha A^{H}\left(A{\hat{m}}^{t}-y\right)$$

And a neural network‐based regularization step

(9) $${\hat{m}}^{t+1}=D_{\phi }\left(z^{t+1}\right)$$

where this work adopted a CNN of a ResNet‐like architecture (139 680 trainable parameters, number of layers = 5) for $D_{\phi }$. As phase information is vital for PC‐MRI, a fully complex neural network was used instead of separating real and imaginary channels. The activation function was ℂReLU=ReLU(a)+i·ReLU(b) for complex data d=a+ib, and the complex convolution W*d=(X*a−Y*b)+i(Y*a+X*b) for complex kernel W=X+iY. Six unrolls of DC‐CNN layers were used, where the CNN shared weights in each unroll.

The learnable parameters include ($k_{\mathrm{yc}},k_{\mathrm{zc}}$, $s_{r}$, θ) for the wave readout and $\left(k_{\mathrm{yc}},k_{\mathrm{zc}}\right)$ for the no‐wave readout, step size α, and DL denoiser parameters ϕ. These parameters were trained by minimizing the image domain NRMSE loss between the reconstructed image $\hat{m}$ and the pseudo‐fully sampled images m: $\text{loss}=\sqrt{\sum {\left|m-\hat{m}\right|}^{2}}/\sqrt{\sum {\left|m\right|}^{2}},\;m,\hat{m}\in \mathbb{C}$. Adam optimizer (learning rate = 1e−3, weight decay = 1e−3) was used. The helix center coordinates $\omega_{c}$ in the wave and no‐wave cases was initialized with identical variable density Poisson disc sampling. Models were trained on a V100 GPU and converged at around 140 h based on validation loss convergence. The training script and trained models are provided on GitHub (https://github.com/uwmri/LearnedWave).

### 2.4 Special Considerations for Training

#### 2.4.1 Accurate Gradient Tracking

As the NUFFT operator is an approximation, errors in the gradients with respect to the sampling coordinates ω will accumulate over training epochs using native auto‐differentiation in DL frameworks such as PyTorch and Tensorflow to track gradients even with differentiable implementation of NUFFT [46]. To avoid this, custom gradients were written for the backpropagation chain. As the loss function $L\left(\omega \right)={\left|\left|\hat{m}-m\right|\right|}_{2}^{2}:\mathbb{C}\to \mathbb{R}$, Wirtinger calculus allows us to simplify the chain rule equation for the backward pass of the encoding operator A w.r.t the coordinates ω:

(10) ∂L∂ω=∂L∂(Am)**∂(Am)∂ω*+∂L∂(Am)*∂Am∂ω*=∂L∂y**∂y∂ω*+∂L∂y*∂y∂ω*=2Re∂L∂y*∂y∂ω**

where *H* denotes Hermitian transpose and * denotes conjugate.

Similarly, for the backward pass of $A^{H}$ w.r.t ω:

(11) ∂L∂ω=∂L∂AHy**∂AHy∂ω*+∂L∂AHy*∂AHy∂ω*=∂L∂m**∂m∂ω*+∂L∂m*∂m∂ω*=2Re∂L∂m*∂m∂ω**

$\frac{\partial L}{\partial m^{*}}$ and $\frac{\partial L}{\partial y^{*}}$ in Equations (10) and (11) can be tracked as the grad output by PyTorch [36], while $\left(\frac{\partial y}{\partial \omega^{*}}\right)=\left(\frac{\partial \mathrm{Am}}{\partial \omega }\right)$ is the Jacobian of A w.r.t. ω, and $\left(\frac{\partial m}{\partial \omega^{*}}\right)=\left(\frac{\partial A^{H}y}{\partial \omega }\right)$ is the Jacobian of $A^{H}$ w.r.t. ω. The two Jacobians have been previously derived by Gossard et al. [47] and Wang et al. [46]. The implementation of the equations above is provided on GitHub (https://github.com/uwmri/LearnedWave) using SigPy's implementation of GPU‐enabled NUFFT with a Kaiser‐Bessel interpolation kernel of width=4 and an oversampling factor of 1.25 [48].

#### 2.4.2 Memory Saving Techniques

Blockwise learning [49] was utilized to avoid memory limitations associated with the use of fully 3D networks in model based deep learning, highlighted in the blue box in Figure 1. In blockwise learning, images were divided into overlapping blocks before feeding into the denoiser and were put back to full volume after, for the next data consistency step. This alone only does not reduce memory requirements as latent images still must be saved in memory for backpropagation. Instead of saving the intermediate latency space images, the denoiser uses gradient checkpointing to only store the input to the denoiser. This reduces memory usage at the cost of increasing computation time.

#### 2.4.3 Generalizability of Background Phase Variation

The background phase in the pseudo‐ground truth data is inherently flat as it was acquired with 3D radial trajectory and coil sensitivities were derived from low resolution data, removing a significant portion of the background phase. As this may not be the case for the prospective wave scans, random first order polynomial phase $\phi =a_{1}x+a_{2}y+a_{3}z+a_{4}$ was added during training $m_{\text{augmented}}=m\cdot e^{2\pi \mathrm{i\phi}\cdot b}$. $a_{i},\left(\;i=1,\ldots ,4\right)$ are random numbers from −10 to 10 and b is a random number ranging 0.3–3. Random complex gaussian noise was also added to complex k‐space data of the pseudo‐ground truth to improve the generalizability of the model:

(12) $$\begin{array}{cc} {\text{kdata}}_{\text{trut}h_{\text{noisy}}} & =Re\left(\text{kdat}a_{\text{truth}}\right)+\text{Gaussian}\left(0,{\left(n\cdot \sigma_{\mathrm{re}}\right)}^{2}\;\right)\\ & \;+i\cdot \left[Im\left(\text{kdat}a_{\text{truth}}\right)+\text{Gaussian}\left(0,{\left(n\cdot \sigma_{\mathrm{im}}\right)}^{2}\right)\right] \end{array}$$

where n is a random number ranging 0.5–1.5, $\sigma_{\mathrm{re}}$ and $\sigma_{\mathrm{im}}$ are estimated noise level of the original pseudo‐ground truth images, which is the median of the k‐space data at the edge of k‐space. The range of the random numbers were tuned heuristically by visually examining the image.

### 2.5 Flow Phantom and In Vivo Scans

To provide prospective evaluation, learned wave, learned no‐wave, and reference 3D radial PC‐MRI scans were collected in a flow phantom and in healthy human participants (*n* = 12, age 23–38 years, mean = 28.8 year) on a 3.0T scanner (GE Signa Premier). For phantom scans, a 30‐channel coil anterior array (Air Coil, GE Healthcare) was used in addition to a built‐in posterior array while in vivo scans used a 48‐channel head coil. Identical scan parameters were used for both in vivo and phantom scans: TE/TR = 3.4/7.7 ms, *V*_enc_ = 80 cm/s, FOV = 220 × 220 × 220 mm^3^, resolution = 0.86 mm isotropic, 4385 phase encodings (*R* = 11.2), echo fraction = 0.75, scan time 2.25 min. The acceleration was chosen such that the scan time roughly matches that of the 3D PC‐MRA scan used in the “Fast Stroke” protocol at our institute [50]. The scan parameters for the reference scan are listed in Section 2.1. A short 3D radial scan with scan time matching the wave and no‐wave scan were also collected for comparison. Both 3D radial scans were reconstructed with an L1‐wavelet reconstruction as described in Section 2.1. The wave and no‐wave scans were reconstructed with the same MoDL learned during their respective training.

**FIGURE 2:**
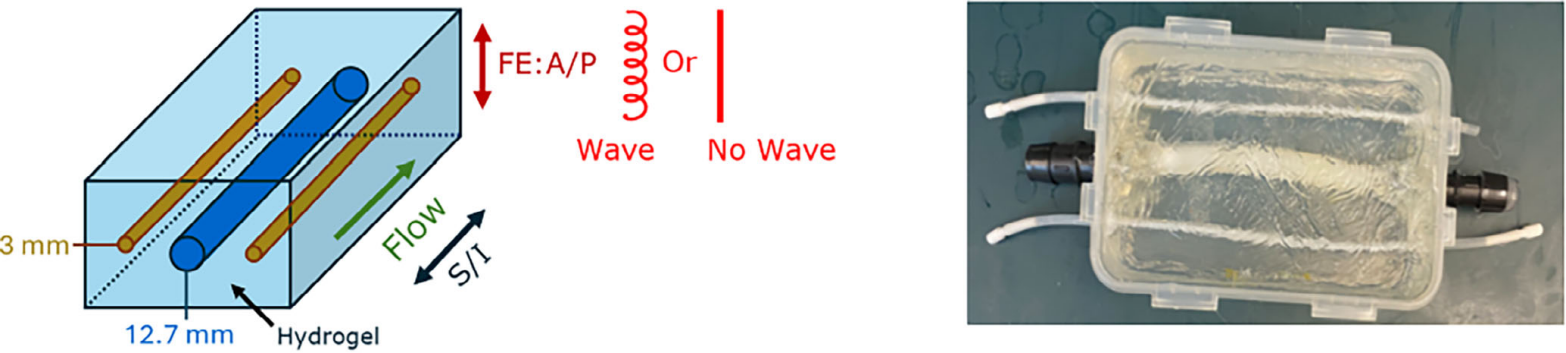
Illustration and photo of the flow phantom, which consist of three tubes embedded in hydrogel. The tubes are connected to a pump and compliance chamber outside of the scanner room.

To evaluate quantitative performance, flow phantom experiments were performed. The flow phantom consists of two small tubes of diameter = 3 mm and a larger tube of diameter = 12.7 mm in a phantom box filled with hydrogel. The hydrogel material is fabricated by dissolving a base solution consisting of acrylamide and bis‐acrylamide (Fisher Scientific) and de‐ionized (D.I.) water. Cross‐linking of the gel was initiated by the addition of Tetramethylethylenediamine (TEMED) and ammonium persulfate (Fisher Scientific). The phantom was connected to a MR‐compatible flow circuit and a pump capable of reproducing pulsatile flow (BDC PD‐1100, BCD Laboratories, Wheat Ridge, CO). A compliance chamber (CC) was attached to the outflow tract of the pump and acts as a dampening element, which allows for control of the pressure pulse in the flow circuit and thus, the pulsatile flow waveform delivered to the tubes in the phantom. The system was filled with D.I. water doped with Gadolinium (Gadavist) at 0.1 mmol/kg. A waveform mimicking arterial flow was produced with average flow rates in the smaller tubes at 0.15 and 0.17 L/min and the larger tube at 1.83 L/min, which were confirmed using clamp‐on ultrasonic flow probes (PXL Series, Transonic Systems, Ithaca, NY) (Figure 2).

### 2.6 Analysis

#### 2.6.1 Flow Rate

For the flow phantom, flow rates were measured at the same five points along the large and two small tubes from the learned wave, learned no‐wave, short 3D radial, and reference 3D radial scans and compared to the doppler flow probe measurements.

For the in vivo scans, the intracranial vasculature was segmented using QVT tool [51] and the flow rate was measured at four relatively straight segments of the internal carotid artery (ICA), namely ICA C4‐5, C3, C2, and C1. In each segment, five measurements were taken from consecutive cross sections on the centerline. By conservation of mass, these 20 flow rate measures should be consistent (e.g., no vessel branching between landmarks). The coefficient of variation and two‐way mixed effects intraclass correlation coefficient (ICC) was calculated and averaged over all participants. Pairwise Bland–Altman analysis was performed of the measured flow rates in the three short scans against the reference 3D radial scan (Figure S1).

#### 2.6.2 $v_{\max}$ Variability

Five consecutive cross sections in the four ICA segments and in middle cerebral artery (MCA), basilar artery (BA), and superior sagittal sinus (SSS) were selected, and the maximum velocities ($v_{\max}$) within each cross section were recorded. Without significant vessel changes, $v_{\max}$ should remain similar in consecutive planes, and the main source of variability is noise. Therefore, the standard deviation of $v_{\max}$ ($\sigma_{v_{\max}}$) can be used as a measurement for noise level in the different scans.

#### 2.6.3 Pixelwise Velocity Comparison

Pixelwise velocity comparisons were made in major vessels to compare the learned wave, learned no‐wave, short 3D radial and reference 3D radial scans in both phantom and human participant scans. The two scans being compared were registered to a common space based on magnitude images using rigid registration in MATLAB. Registration to common space ensures the rotation and translation are equally applied to the dataset pair being compared [28]. The mask for major vessels was obtained by applying a threshold at 10% of the max intensity in the complex difference image. Bland–Altman and linear regression analysis were done using pooling from all participants (Figure 8) and individually (Figure S2).

## 3 Results

### 3.1 Sampling Parameters

**FIGURE 3:**
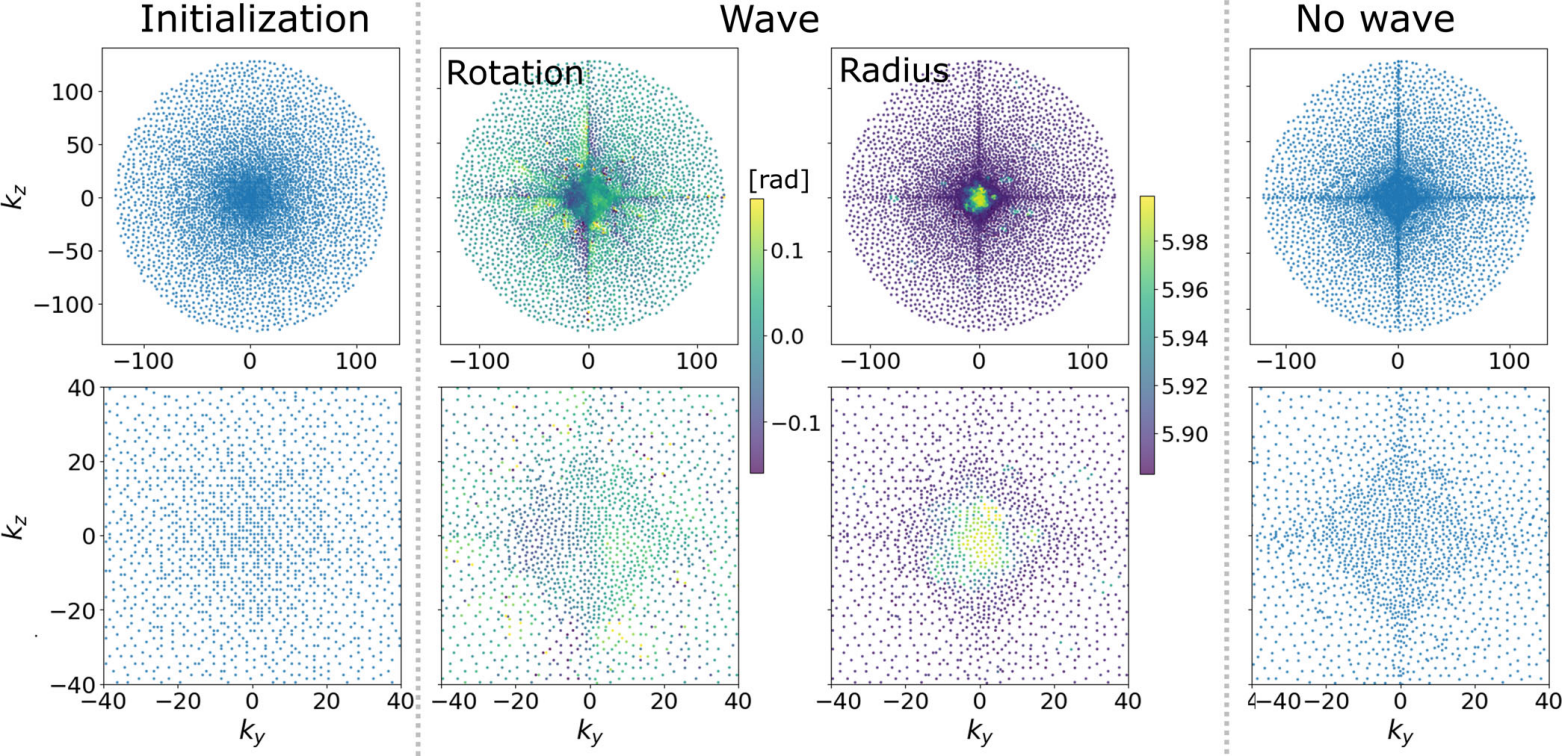
The Poisson disc initialization and the learned sampling patterns with and without wave encoding, and their zoomed‐in views (bottom row). The color in the wave sampling pattern represents the wave radius and rotation angle θ around the helix's long axis.

Figure 3 shows the learned sampling patterns with and without wave encoding. The two patterns are somewhat similar, with the wave encoding having a slightly less densely sampled k‐space center. The sampling points gather along the $k_{y}=0$ and $k_{z}=0$ axes in both cases. The rotation angle of the optimized *θ* roughly has opposite signs around these two axes (Table 1).

**TABLE 1:** Flow rate (L/min) from ultrasound flow probe and the four different MRI scans.

	Flow probe	Wave	No‐wave	Short 3D radial	Reference
Small tube 1	0.17	0.170 ± 0.002	0.166 ± 0.008	0.180 ± 0.006	0.171 ± 0.006
Small tube 2	0.15	0.152 ± 0.003	0.152 ± 0.005	0.160 ± 0.006	0.154 ± 0.002
Large tube	1.83	1.838 ± 0.044	1.834 ± 0.026	1.818 ± 0.031	1.838 ± 0.044

### 3.2 Flow Phantom

**FIGURE 4:**
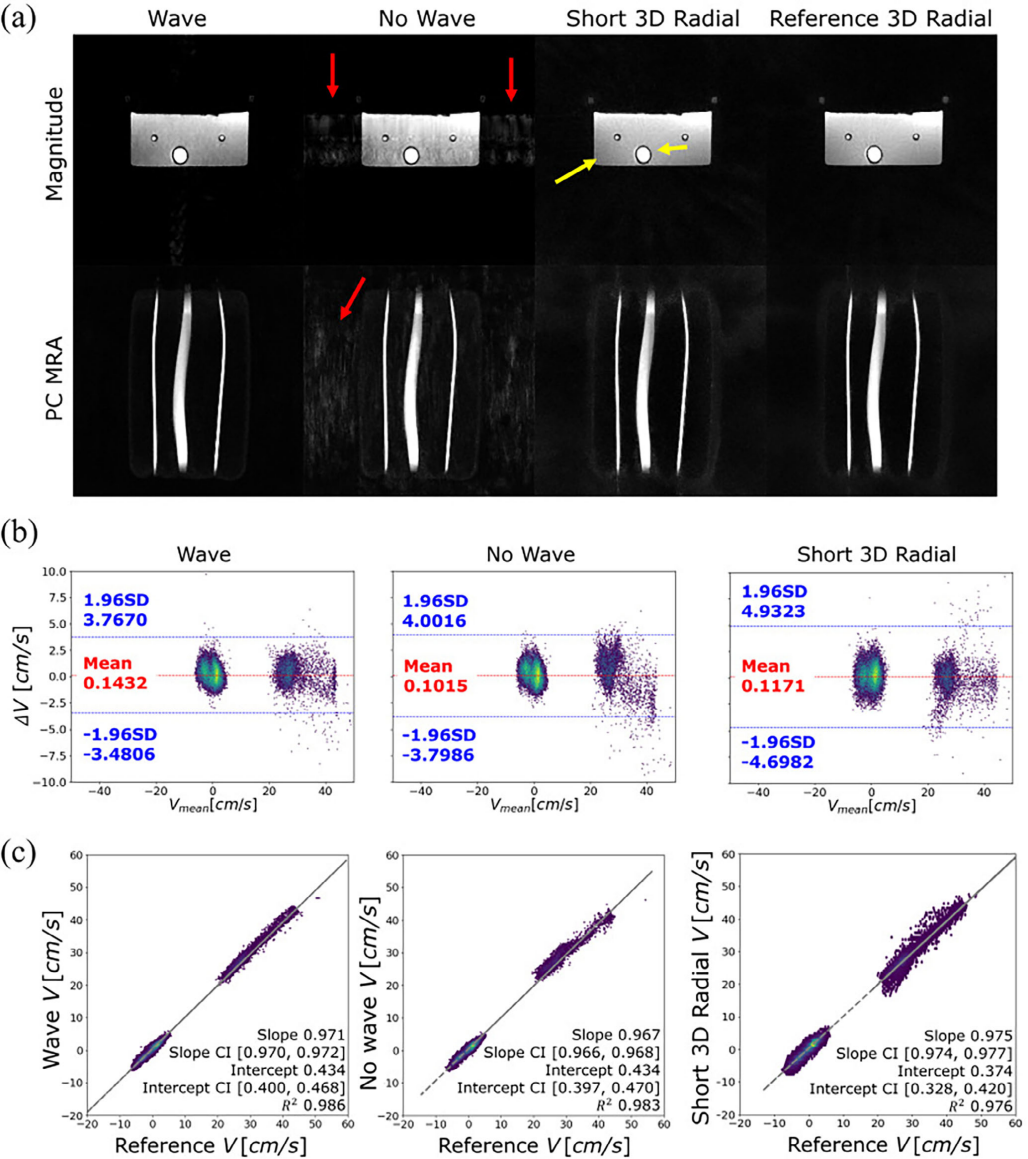
(a) Magnitude and phase contrast angiogram (PC MRA) from learned wave, learned no‐wave, short 3D radial and reference 3D radial scans. Residue aliasing is present in the no‐wave scan (red arrows) and the short 3D radial shows some streaking and blurring (yellow arrow). (b) Bland–Altman and (c) linear regression of the pixelwise velocity analysis in the phantom scans. Overall, the wave scan shows better agreement with the reference 3D radial scan in terms of limits of agreement (1.96 SD), slope, confidence interval (CI), and *R* ^2^, in linear regression despite a slightly higher mean velocity difference. The confidence intervals are for 95% confidence.

As is shown in Figure 4a, in both the magnitude and PC‐MRA images of the flow phantom, the learned wave scan shows the least amount of noise and artifacts among the three accelerated scans. The no‐wave scan showed residual aliasing whereas the short 3D radial showed some streaking and blurring, especially around the edges of the large tube. Pixelwise velocity comparisons of the co‐registered learned wave, learned no‐wave, short 3D radial PC MRI against the reference 3D radial PC MRI are shown in Figure 4b,c. All three short scans show good agreement with the reference scan with *R* ^2^ values of 0.986, 0.983, and 0.976, respectively, with the wave scan being the highest. The confidence interval for the slope and the intercept were the smallest for the wave scan as well, albeit having a slightly lower slope than the short 3D radial scan. Shown in the Bland–Altman plot (Figure 4b, the learned wave scan also had the lowest limit of agreement (LOA) at 3.62 cm/s, with no‐wave at 3.90 cm/s and short 3D radial at 4.82 cm/s despite slightly higher mean difference. Note that as the flow in the tubes are highly directional with mild curvature, the pixel velocities are clustered with $v_{x}$ and $v_{y}$ centered around 0 cm/s while $v_{z}$ ranges from 20 to 40 cm/s. The wave scan showed the least amount of deviation compared to the reference scan in both clusters. All scans show relatively good agreement with the ultrasound flow probe, with the reference 3D radial and learned wave scan performing the best and the short 3D radial performs the worst.

### 3.3 In Vivo Scans

**FIGURE 5:**
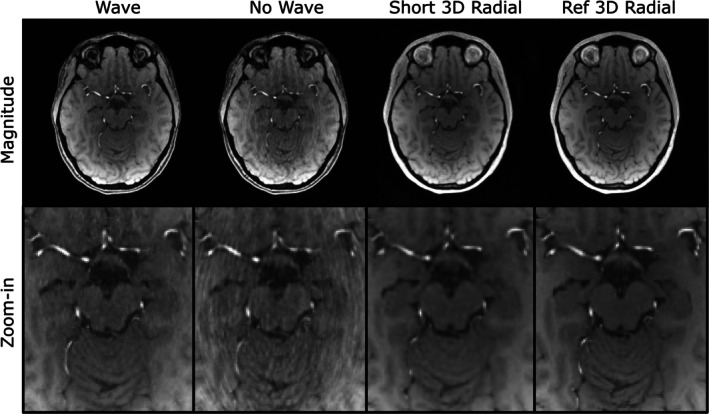
Magnitude images and zoomed‐in views from the wave, no‐wave, short 3D radial, and reference 3D radial scans of a volunteer. The wave scan shows less aliasing and blurring than the no‐wave scan and the short 3D radial scan.

**FIGURE 6:**
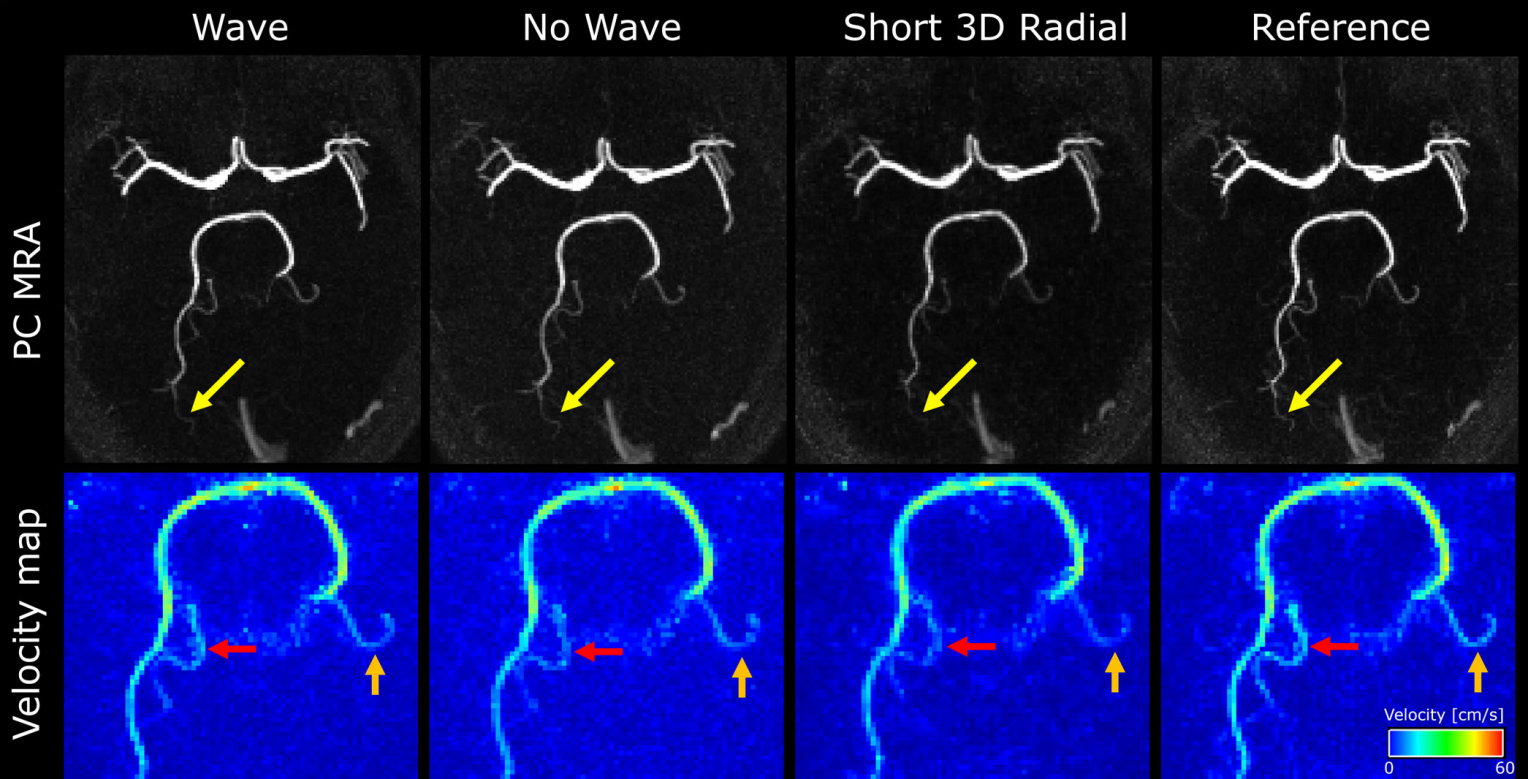
Phase contrast angiogram (PC MRA) and velocity maps from the wave, no‐wave, short 3D radial and reference 3D radial scans of a volunteer. Yellow and red solid arrows highlight that the wave scan provides better small vessel visualization compared to no wave and short 3D radial.

**FIGURE 7:**
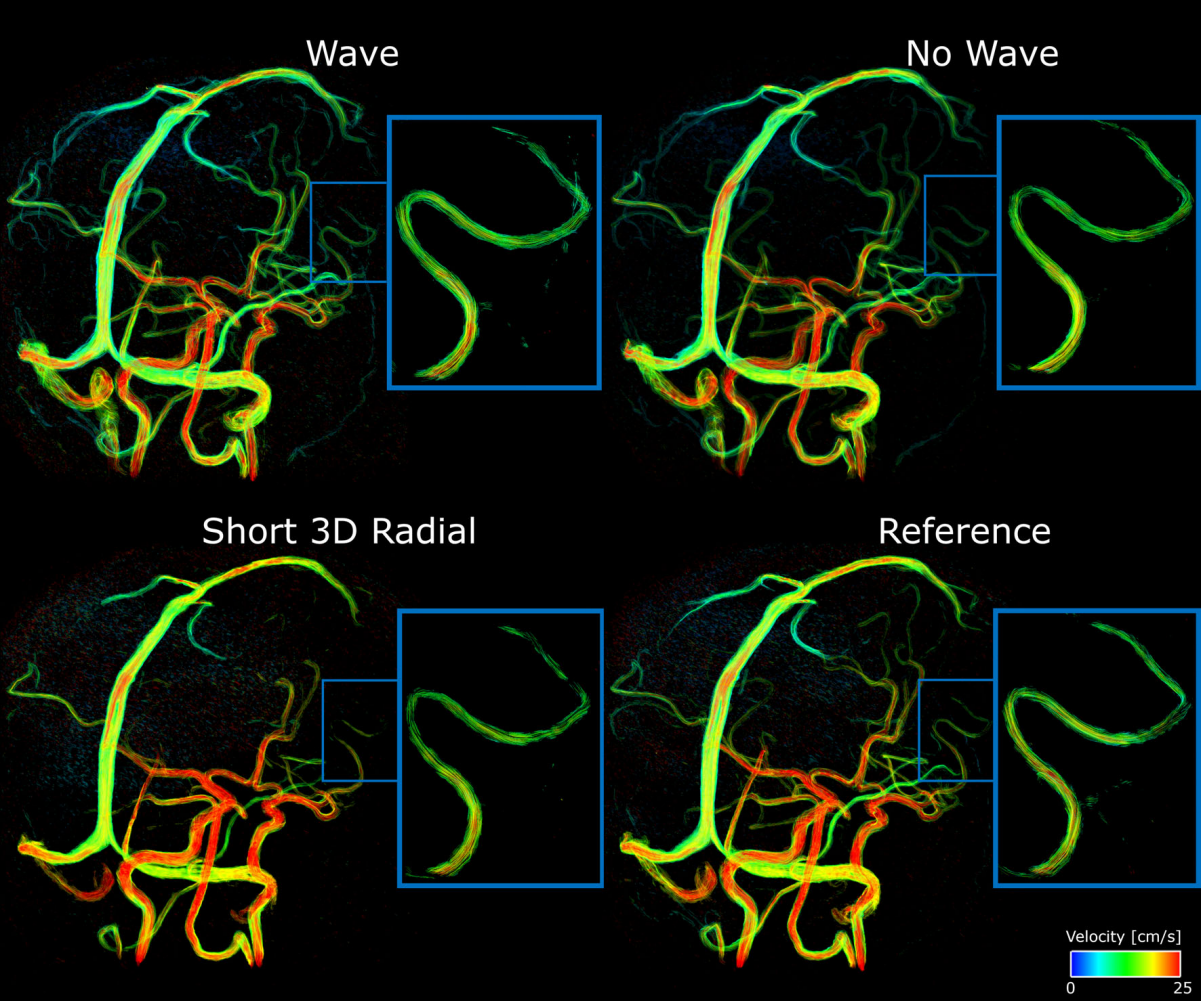
Streamline plots of the whole brain and zoom‐in views of the wave, no‐wave, short 3D radial and reference 3D radial scans of a volunteer. The short 3D radial scan shows the least number of smaller vessels while the wave scan shows even more smaller vessels than the reference scan and depicts lower velocity better despite slightly higher noise level. The blue boxes highlight the cortical segment of the MCA.

Magnitude, velocity, and streamline images from an exemplary human participant are shown in Figures 5, 6, 7. In the magnitude images, the no‐wave scan has substantial residual aliasing, whereas the short 3D radial scan shows visible blurring. In the 8‐slice maximum intensity projection of the PC MRA (Figure 6, top row), both the no‐wave and the short 3D radial scan are blurrier than the wave scan and show lower signal in smaller vessels. In the velocity map (Figure 6, second row), the wave scan shows higher velocity‐to‐noise (VNR) than the no‐wave and short 3D radial scan (arrows). Figures 6 and 7 illustrate that the wave scan produces high quality angiograms and streamlines that are of quality close to the reference scan, despite slightly more background noise visually. The no‐wave scan is blurrier, while the short 3D radial lacks details. The cortical segment (box) is barely visible in the whole brain view of the short 3D radial scan and is the clearest in the wave scan.

**FIGURE 8:**
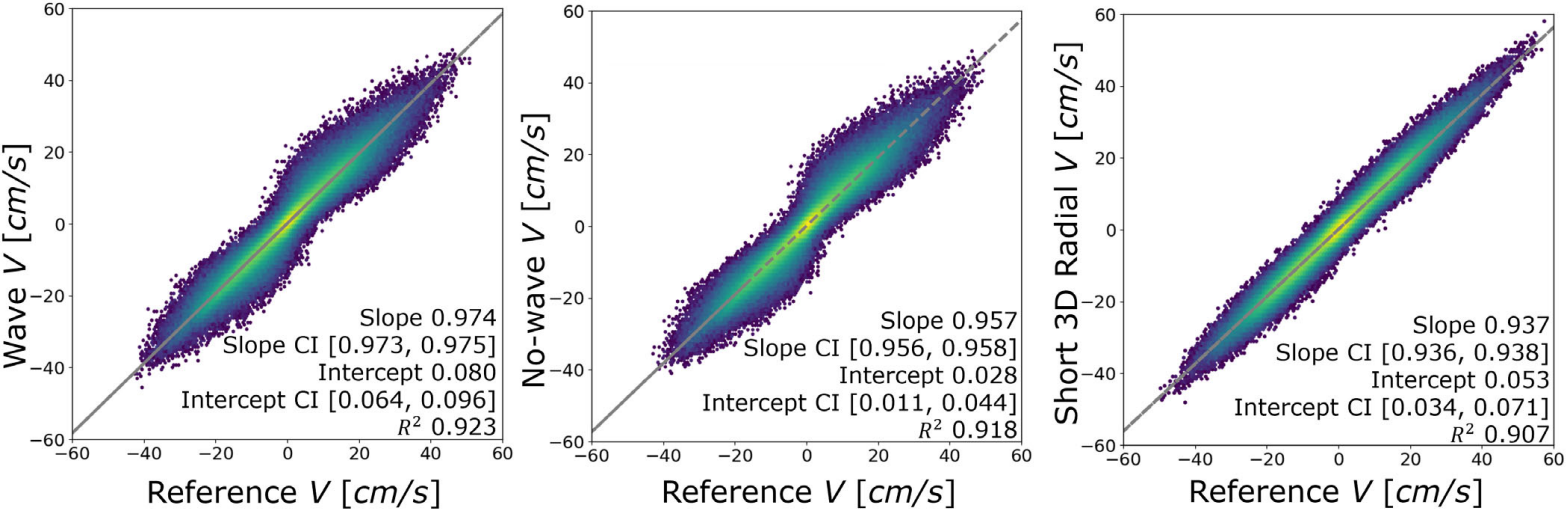
Linear regression analysis of the wave, no‐wave, and short 3D radial scans compared pixelwise to the reference 3D radial scans of all volunteer scans. (b) Bland–Altman analysis of the wave, no‐wave, and short 3D radial scans compared to the reference 3D radial scans, as well as the wave compared to the no‐wave and the wave compared to the short 3D radial scans of all volunteers. All plots were based on images after registration.

Figure 8 shows the correlation of the pixelwise velocity from three shorter scans compared against the reference scan from all volunteers. The wave scan showed the slope closest to 1, at 0.974 ($R^{2}=0.923$), while the short 3D radial tends to underestimate the velocity the most, with a slope of 0.937 ($R^{2}=0.907$). The short 3D radial scan had the largest range in limits of agreement (LoA) of 8.533 cm/s, followed by the no‐wave scan and wave scan at 7.309 and 7.176 cm/s, respectively. The participant‐pooled correlation plots (Figure 8) and Bland–Altman plots of wave/reference and no‐wave/reference (Figure S3) show a “bow‐tie” shape, which was not seen in the flow phantom experiments. This pattern can be observed in some participants more than others (Figure S2). Overall, the wave scan shows better slope, $R^{2}$ and LoA in individuals. The mean agreements fluctuate but are all small for all three short scans.

Conservation of mass verification showed that the flow rates measured in the wave scan from the four ICA segments (five measurements/segment) had the lowest coefficient of variation (CV) among the three short scans at 6.569, which is very close to the reference scan (6.553), whereas the no‐wave and short 3D radial scans show CVs of 7.431 and 6.718, respectively. The wave scan also provided the highest consistency of the four methods with an ICC of 0.927, compared to the reference, no‐wave, and short 3D radial, being 0.910, 0.904, and 0.913, respectively. The Bland–Altman analysis on the ICA flow rate (Figure S1) revealed that the wave scan provided a closer measurement to the reference, with mean = 0.004 L/min and range of LoA = 0.058 L/min, compared to the no‐wave scan (mean = 0.007 L/min, range of LoA = 0.064 L/min).

**FIGURE 9:**
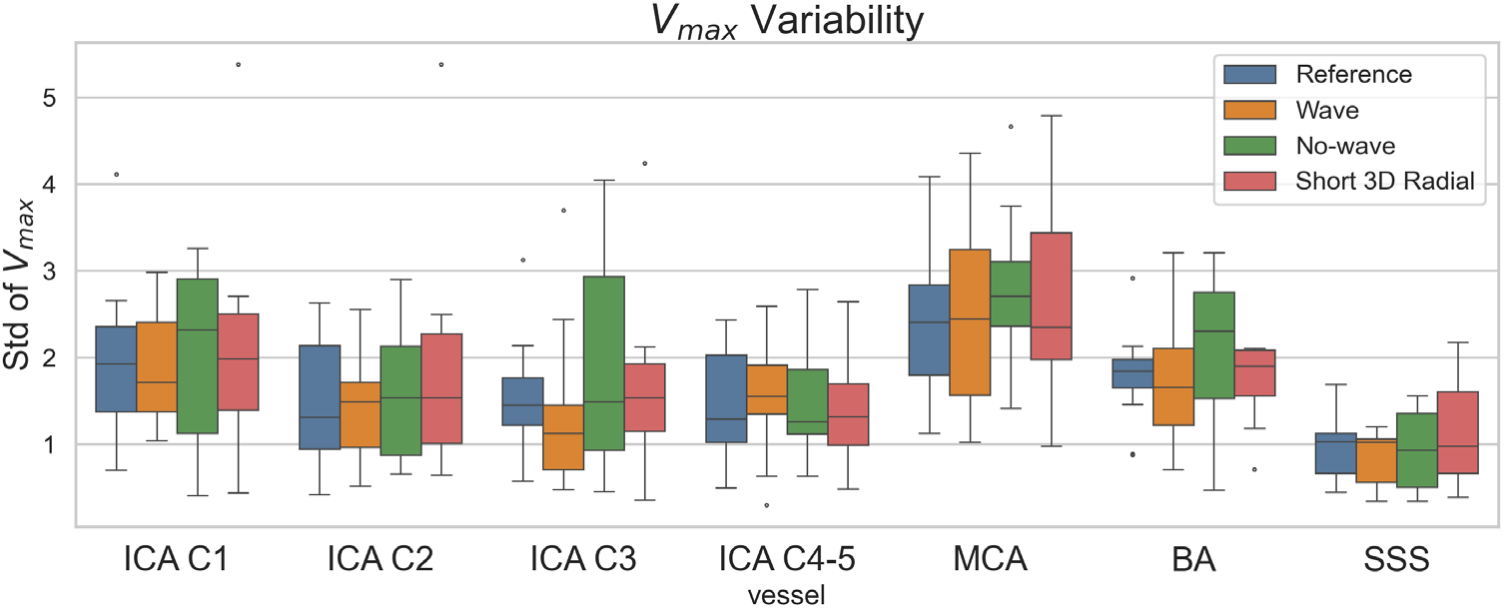
Variability of. $v_{\max}$ in four ICA segments, MCA, BA, and SSS in the reference and three short scans.

For $v_{\max}$ variability (Figure 9), the wave scan shows the lowest $\sigma_{v_{\max}}$ amoung the three short scans in MCA, BA, SSS, and most ICA segments, with the exception of ICA C4‐5.

## 4 Discussion

This work investigated the feasibility of learning sampling patterns and wave encoding parameters directly from data for the application of 3D PC‐MRI, with a DL reconstruction learned jointly. We prospectively evaluated the image quality and flow measurements accuracies in a flow phantom and in healthy human participants. Despite being trained on human data and on a different population (mean age = 32/66.8 years, healthy volunteers/WADRC clinical subjects) than the prospective scans (mean age = 28.8 years healthy volunteers), the learned sampling pattern with and without wave readout produced high quality angiogram and accurate flow measurements in the flow phantom and in vivo compared to the doppler flow probe and the reference scan. Both DL based methods outperformed 3D radial scans with matching scan time, except for lower consistency of in vivo flow rate measurement in the no‐wave scan. Here, the potential use of learned wave sampling using data driven learning is demonstrated, providing evidence that DL aided sampling and reconstruction may outperform 3D radial sampling via more robust and faster imaging. Wave encoding resulted in higher image quality and more accurate flow measurements compared to no‐wave straight line sampling, proving the value of optimizing 3D trajectories as opposed to 2D sampling patterns.

Non‐Cartesian imaging such as 3D radial [15, 16] and spiral [52] has long provided acceleration strategies for flow imaging. However, these methods are particularly sensitive to off‐resonance blurring, system imperfections, and require lengthy imaging reconstructions. At the same time, DL denoising [28] and super‐resolution [53] have been proposed to reduce the scan time and improve the test–retest reproducibility. However, the work of Jolicoeur et al. [28] focused on flow measurements in major vessels and the work of Shit et al. [53] was not validated prospectively. In this work, we aimed to combine the benefits from non‐Cartesian sampling efficiency and DL. Using joint learning of wave encoding parameters, sampling patterns, and MoDL reconstruction, high quality and accurate 3D PC MRI scans were acquired in just over 2 min and can be reconstructed in ˜8.6 min (single NVIDIA Quadro P6000). Such, rapid 3D PC MRI scanning provides the potential to substantially improve the accessibility to 3D PC MRI for clinical screening and evaluation. In such applications, rapid acquisition and reconstruction is crucial for identifying stenosis, occlusion or hemorrhage and the time‐resolved information provided by 4D flow may not outweigh the longer scan, reconstruction and post‐processing time. For example, University of Wisconsin uses a “Fast Stroke” screening protocol [50] with a 2‐min 3D PC MRA. However, the images produced by the current protocol are relatively low resolution (1.2 × 1.6 × 1.8 mm^3^) and only sufficient to identify gross malformations. The resolution provided by the learned wave scan (0.86 mm isotropic) enables the identification of smaller lesions and the quantification of time‐averaged flow without extra time cost.

In this work, we adopted the Jacobian matrices calculation by Wang et al. [46] and Gossard et al. [47] for the tracking loss with respect to sampling coordinates through NUFFT and expanded it to be compatible with wave parameters optimization and block‐wise MoDL. Using Jacobians allows more accurate backpropagation and potentially avoids suboptimal solutions due to error accumulation. However, whether the differences is perceivable by human readers in the end images produced by using Jacobians versus relying on the built‐in auto‐differentiation mechanism in PyTorch/Tensorflow [35] for gradient tracking remains to be investigated.

The application of DL to 3D PC MRI requires some special consideration. In training our framework, aiming to preserve the phase information, we did not treat the real and imaginary parts as separate channels. We initially used the same real‐valued convolution kernel on the real and imaginary parts separately, which is a common approach [21, 54, 55]. However, this caused artifacts at the denoiser step with patterns similar to the phase image (Figure S4). This could be due to the prospective scans having different phase conditions than the training and the true complex convolution is more robust against such variations [56]. True complex convolution is more computationally expensive and GPU memory demanding, therefore, it may be worth exploring if the robustness can be achieved via other means, for example, adding phase information in the loss function. What is also worth noting is that for applications where fully sampled data is impractical to collect, the quality of the “ground truth” may heavily influence the results. In our case, the standard reconstruction method for the 3D radial scans is a NUFFT followed by optimized coil combination (PILS) [57], utilized due to its quick reconstruction time. Visually, these images are of similar quality to images reconstructed using compressed sensing, however, they have missing lines in k‐space. Training on PILS reconstructed images results in a sampling pattern similar to 2D radial sampling (Figure S5a).

Another interesting observation was the dependency of the learned sampling pattern on the coupled reconstruction method. When using PILS [57] reconstruction, the final converged learned sampling pattern appears to be spatially uniform (Figure S5b). When switching to MoDL, we observed a pileup of sampling points near the $k_{y}$ and $k_{z}$ axes, which might be due to the DL recon learned the discrete nature of the image and therefore place high significance on sampling around the axes. Opposite rotations near the axes might be attributed to this as well. Thorough investigation on how the complexity of the network, how other reconstruction algorithms or how different anatomies would affect the learned sampling pattern remain to be a potential direction worth pursuing in the future.

In the pixelwise velocity analysis of the in vivo data pooled from all volunteers (Figures 8 and S3), there is a “bow‐tie” shape in the wave versus reference and no‐wave versus reference plots, whereas it is less prominent in the short 3D radial versus reference plot. This might be attributed to the reference and short 3D radials scans been more similar in terms of image texture, artifacts, noise, geometry, and response to motion as they share the same trajectory. For example, off‐resonance will lead to a non‐uniform shift in wave but a blurring in 3D radial. These characteristics would affect the quality of rigid registration. As a test, we performed registration of wave/short 3D radial, and wave/no‐wave scans and performed Bland–Altman analysis. (Figure S3) and the wave versus no‐wave plot shows less of the “bow‐tie” shape than the wave‐reference and wave‐short 3D radial, supporting the hypotheses that these effects are due to spatial misregistration. Moreover, in the well‐controlled flow phantom studies, no such shape was observed. Also, in our protocol scans were collected sequentially: reference 3D radial, short 3D radial, wave, and no‐wave scan. There has been evidence suggesting inter‐scan physiologic changes can cause such “bow‐tie” shape [28] and we have also noticed more inter‐scan motion between the reference/wave and reference/no‐wave than the reference/short 3D radial scans.

One limitation of this work is the lack of fully sampled data. As is indicated in Figure S5a, the quality of “ground truth” data affects the learned sampling pattern, and therefore the learned sampling is tuned to the pseudo‐ground truth data. As obtaining relatively fully sampled ground truth 4D flow data is extremely challenging, self‐supervised learning would be preferred. However, its extension to sampling coordinates optimization in non‐obvious. Another limitation is that we only investigated time‐averaged sampling. Extending our work to time‐resolved flow imaging (i.e., 4D Flow) is challenging both computationally and practically. Computationally, 4D sampling would require a ˜10–20x increase in GPU memory and computation time. Combining spatially optimized sampling pattern with a heuristic temporal sampling strategy that provides uniform coverage robust to temporal binning could be one way and require further investigation.

## 5 Conclusion

Jointly learned non‐Cartesian sampling with wave encoding and DL reconstruction provided high quality 3D PC MRA in just over 2 min. Flow measurements were proven to be accurate in phantom and in vivo prospectively, providing evidence for the value of DL in quantitative MR. The presented technique has strong potential in clinical translation, especially for screening. It also serves as a foundation for future exploration in 4D flow.

## Funding

This work was supported by National Institutes of Health (1R21NS125094, P30AG062715, R01AG021155, R01AG027161, and R01AG075788).

## Conflicts of Interest

GE Healthcare provides research support to the University of Wisconsin.

## Supporting information


**Figure S1**. Bland–Altman plot of the flow rate measured in four ICA segments from all volunteers from the wave, no‐wave and short 3D radial scans compared against the reference scan.
**Figure S2**. Bland–Altman and correlation plots of pixelwise velocity comparisons between the wave, no‐wave, short 3D radial scans, and the reference scan for each individual volunteer.
**Figure S3**. Bland–Altman plots of pixelwise velocity comparisons between the wave/reference, no‐wave/reference, short 3D radial scans/reference, wave/no‐wave and wave/short 3D radial scans with data pooled from all volunteers (*n* = 12).
**Figure S4**. MoDL with separate convolution kernels for real and imaginary parts were able to reconstruct magnitude images of quality similar to MoDL using true complex convolution but led to stripe artifacts in the CD MIP angiogram. The stripes seem to be phase related. This artifact was mitigated when using a fully complex convolution.
**Figure S5**. (a) Learned sampling pattern when trained with PILS reconstructed ground truth images. As PILS does not fill the k‐space well, the sampling pattern converged to the points where data was actually acquired, which goes against the goal of finding the optimal sampling. (b) Learned sampling pattern when using a simple inverse NUFFT with coil‐combine for reconstruction.
**Figure S6**. The theoretical helix trajectory (green) calculated by Equations ((1), (2), (3)) and parameterized by optimal‐time gradient method [1], the trajectory pushed onto the hardware before execution (blue), with deviations from the theoretical due to rounding errors and the actual helix trajectory (red) measured using thin slices gradient calibration method [2].

## Data Availability

The data that support the findings of this study are available on request from the corresponding author. The data are not publicly available due to privacy or ethical restrictions.
