# Supplementary material for: Jointly Learned 3D Non‐Cartesian Sampling With Wave Encoding and Reconstruction for Neurovascular Phase Contrast MRI
Source: Magn Reson Med. 2025 Dec 8;95(5):2554–67. doi: 10.1002/mrm.70215 (PMC12962207; doi:10.1002/mrm.70215)
Supplement: Supplementary file 1 — Figure S1. Bland–Altman plot of the flow rate measured in four ICA segments from all volunteers from the wave, no‐wave and short 3D radial scans compared against the reference scan. Figure S2. Bland–Altman and correlation plots of pixelwise velocity comparisons between the wave, no‐wave, short 3D radial scans, and the reference scan for each individual volunteer. Figure S3. Bland–Altman plots of pixelwise velocity comparisons between the wave/reference, no‐wave/reference, short 3D radial scans/reference, wave/no‐wave and wave/short 3D radial scans with data pooled from all volunteers (n = 12). Figure S4. MoDL with separate convolution kernels for real and imaginary parts were able to reconstruct magnitude images of quality similar to MoDL using true complex convolution but led to stripe artifacts in the CD MIP angiogram. The stripes seem to be phase related. This artifact was mitigated when using a fully complex convolution. Figure S5. (a) Learned sampling pattern when trained with PILS reconstructed ground truth images. As PILS does not fill the k‐space well, the sampling pattern converged to the points where data was actually acquired, which goes against the goal of finding the optimal sampling. (b) Learned sampling pattern when using a simple inverse NUFFT with coil‐combine for reconstruction. Figure S6. The theoretical helix trajectory (green) calculated by Equations ((1), (2), (3)) and parameterized by optimal‐time gradient method [1], the trajectory pushed onto the hardware before execution (blue), with deviations from the theoretical due to rounding errors and the actual helix trajectory (red) measured using thin slices gradient calibration method [2]. [file MRM-95-2554-s001.docx]

**Supporting Information**

1. **
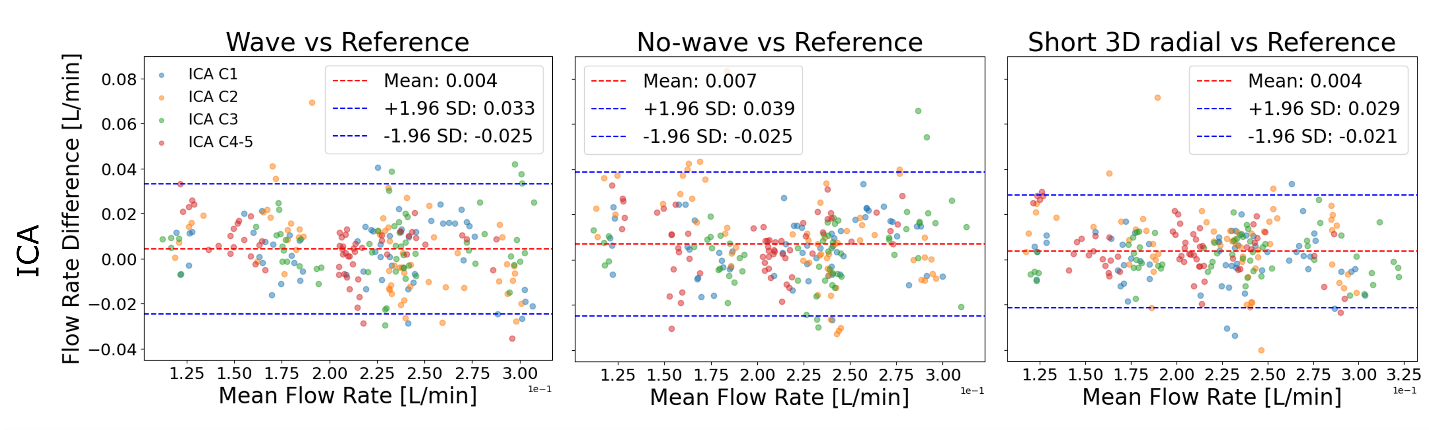
ICA flow rate**

**Figure S1**. Bland-Altman plot of the flow rate measured in four ICA segments from all volunteers from the wave, no-wave and short 3D radial scans compared against the reference scan.

1. **
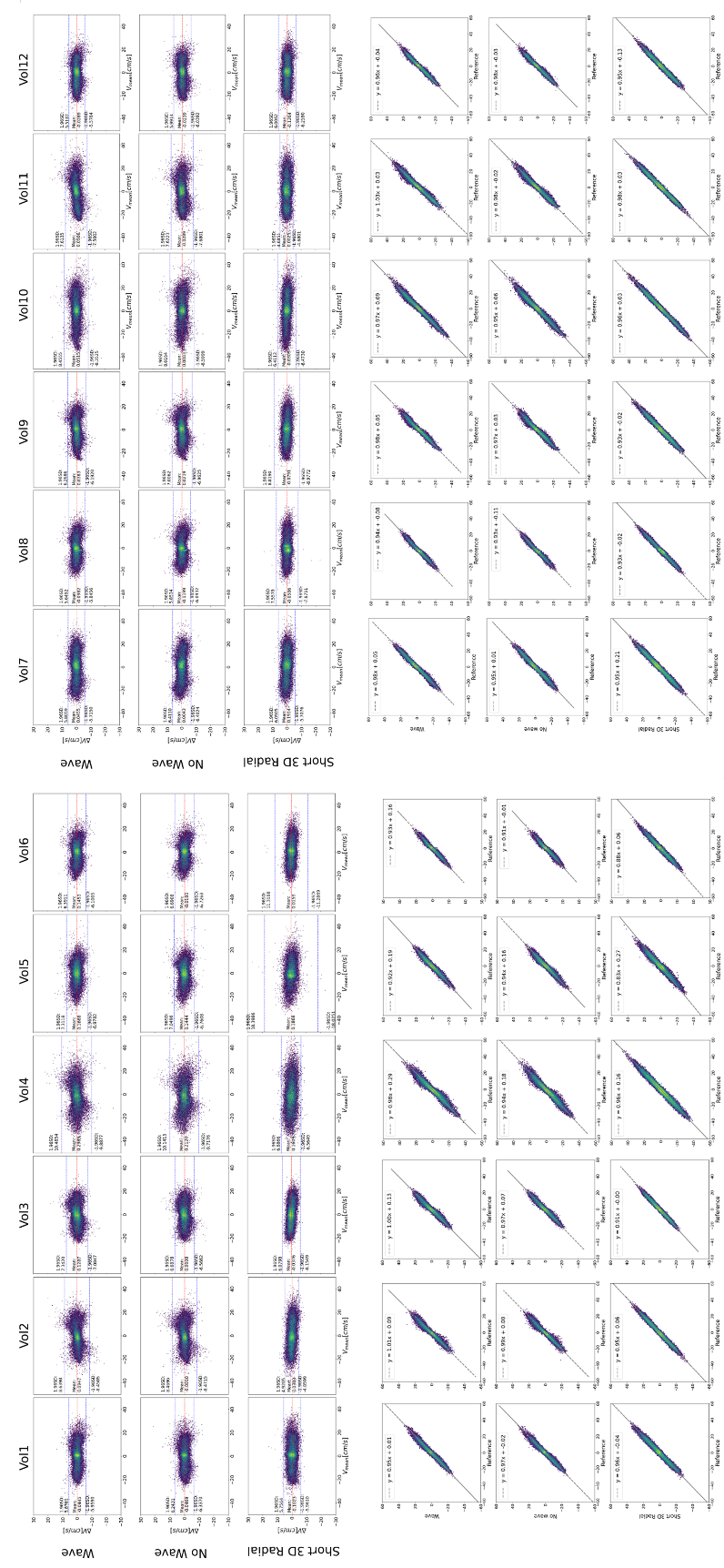
Pixelwise velocity Bland-Altman and correlation plot for each volunteer**

**Figure S2.** Bland-Altman and correlation plots of pixelwise velocity comparisons between the wave, no-wave, short 3D radial scans, and the reference scan for each individual volunteer.

1. **
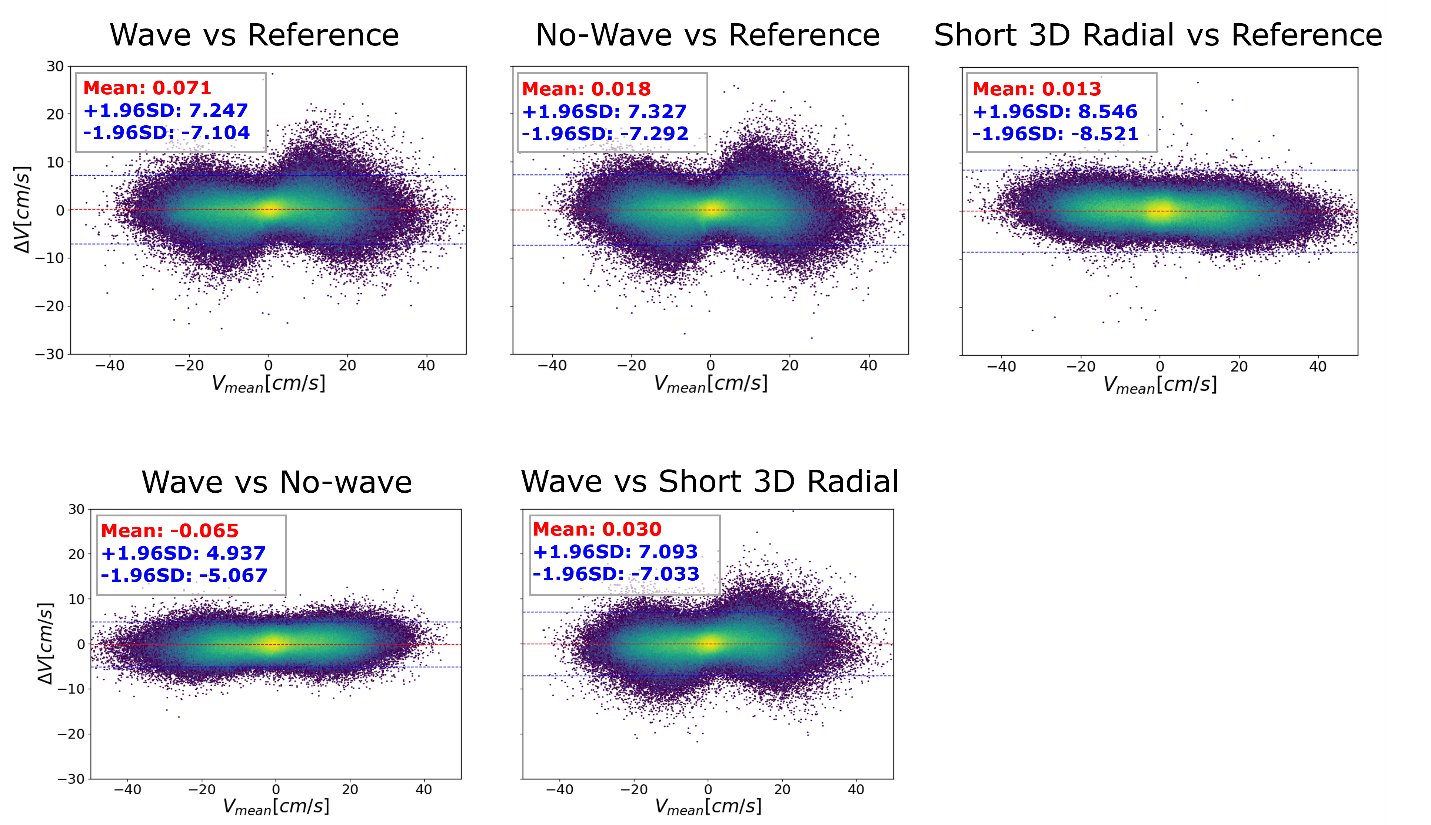
Pixelwise velocity Bland-Altman plot for all volunteers**

**Figure S3.** Bland-Altman plots of pixelwise velocity comparisons between the wave/reference, no-wave/reference, short 3D radial scans/reference, wave/no-wave and wave/short 3D radial scans with data pooled from all volunteers (n=12).

1. **
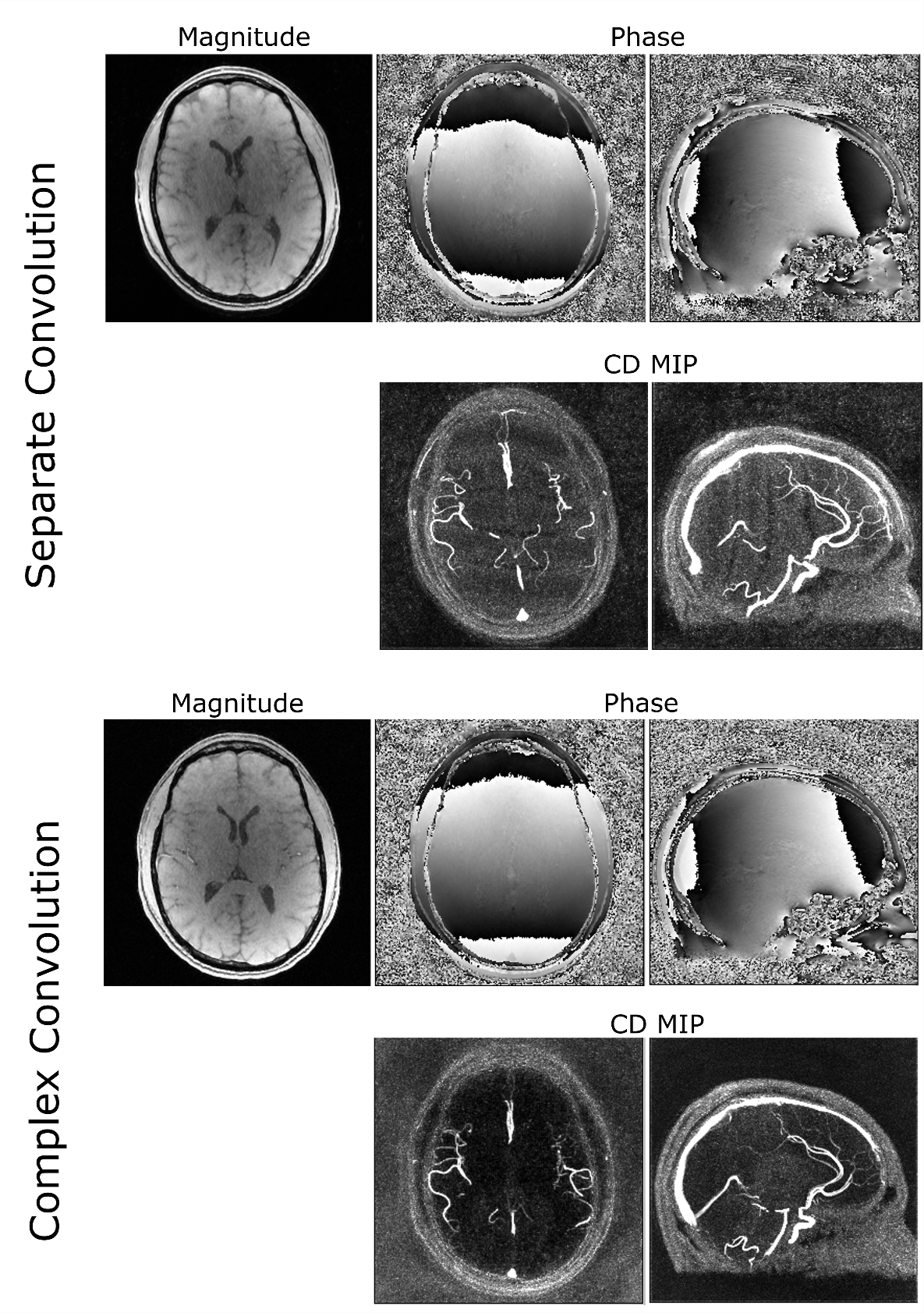
Effects of separate/complex convolution kernel**

**Figure S4.** MoDL with separate convolution kernels for real and imaginary parts were able to reconstruct magnitude images of quality similar to MoDL using true complex convolution but led to stripe artifacts in the CD MIP angiogram. The stripes seem to be phase related. This artifact was mitigated when using a fully complex convolution.

1. **Sampling patterns affected by reconstruction and ground truth data**

**
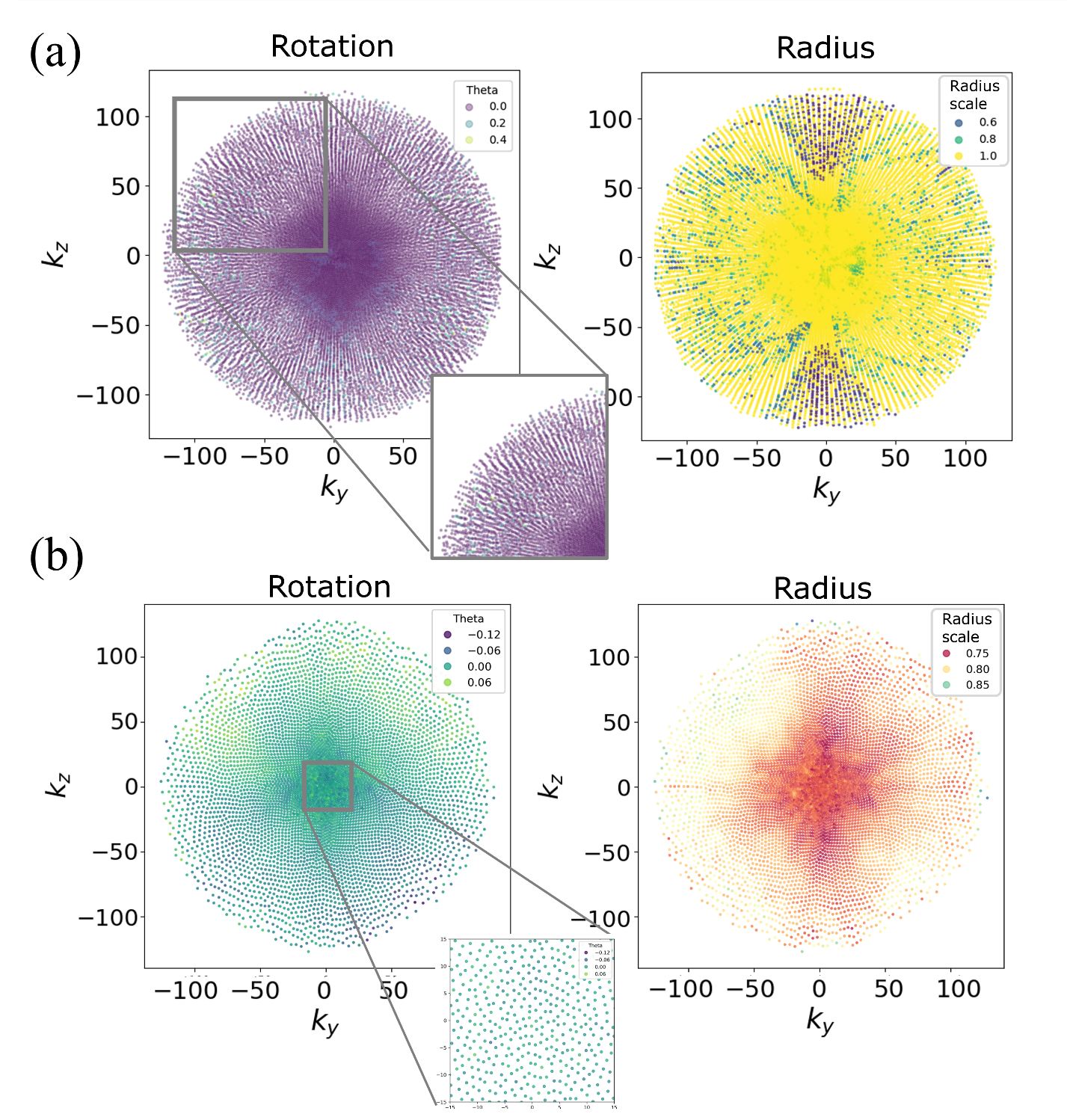
**

**Figure S5.** (a) Learned sampling pattern when trained with PILS reconstructed ground truth images. As PILS does not fill the k-space well, the sampling pattern converged to the points where data was actually acquired, which goes against the goal of finding the optimal sampling. (b) Learned sampling pattern when using a simple inverse NUFFT with coil-combine for reconstruction.


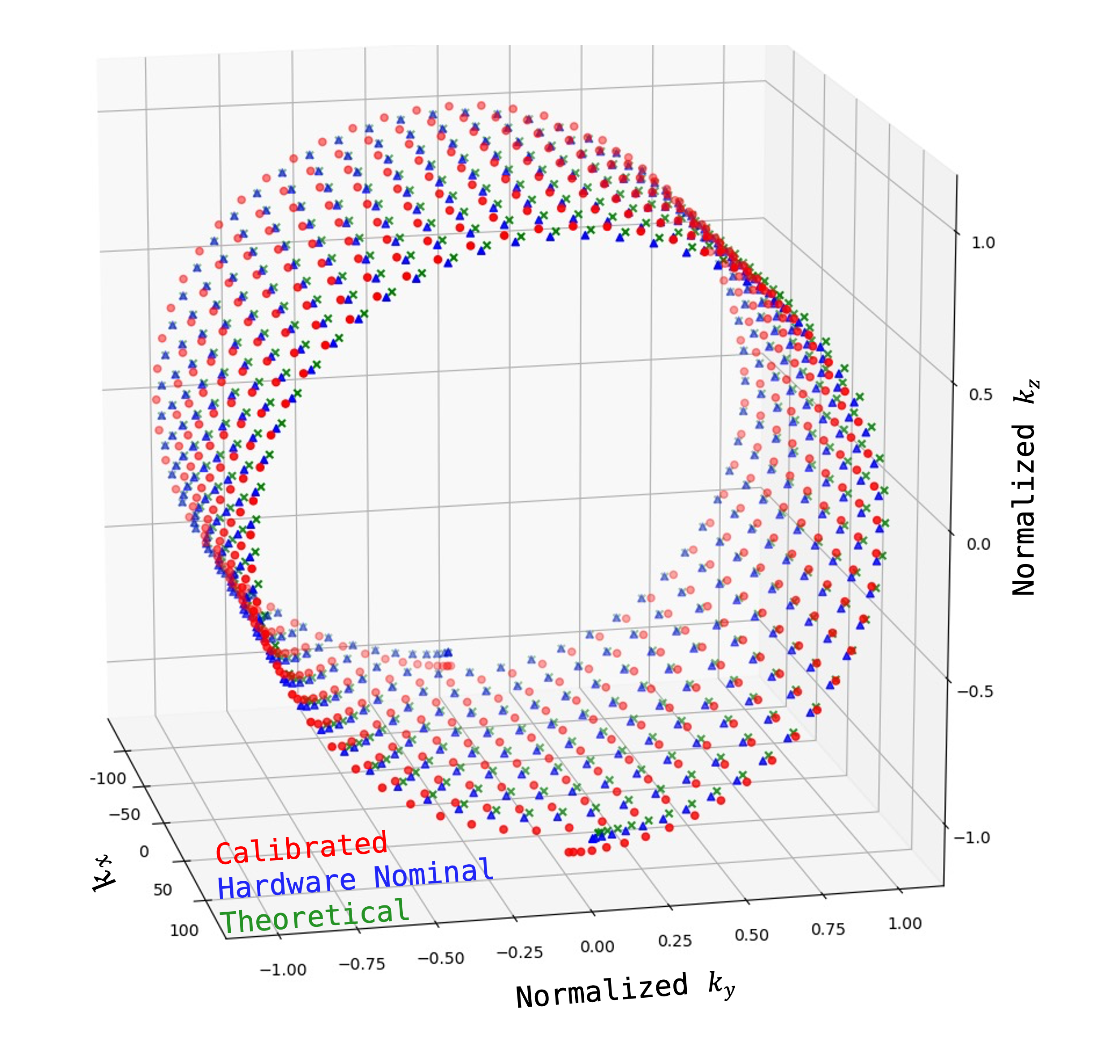


**Figure S6.** The theoretical helix trajectory (green) calculated by Eq.(1-3) and parameterized by optimal-time gradient method^1^, the trajectory pushed onto the hardware before execution (blue), with deviations from the theoretical due to rounding errors and the actual helix trajectory (red) measured using thin slices gradient calibration method^2^.
